# Adsorption of bio-organic eco-corona molecules reduces the toxic response to metallic nanoparticles in *Daphnia magna*

**DOI:** 10.1038/s41598-021-90053-5

**Published:** 2021-05-24

**Authors:** Mikael T. Ekvall, Jonas Hedberg, Inger Odnevall Wallinder, Anders Malmendal, Lars-Anders Hansson, Tommy Cedervall

**Affiliations:** 1 Aquatic Ecology, Department of Biology, Ecology Building, Lund University, 223 62 Lund, Sweden; 2 Department of Biochemistry and Structural Biology, Center for Molecular Protein Science, Lund University, P.O. Box 124, 221 00 Lund, Sweden; 3 NanoLund, Lund University, P.O. Box 118, 221 00 Lund, Sweden; 4 Division of Surface and Corrosion Science, Department of Chemistry, School of Engineering Sciences in Chemistry, Biotechnology and Health, KTH Royal Institute of Technology, Drottning Kristinas väg 51, 100 44 Stockholm, Sweden; 5 Surface Science Western, The University of Western Ontario, 999 Collip Circle, London, Ontario N6G 0J3 Canada; 6 Department of Science and Environment, Roskilde University, P.O. Box 260, 4000 Roskilde, Denmark

**Keywords:** Synthetic chemistry methodology, Diagnostic markers

## Abstract

As the use of engineered nanomaterials increases, so does the risk of them spreading to natural ecosystems. Hitherto, knowledge regarding the toxic properties of nanoparticles (NP’s) and their potential interactions with natural bio-organic molecules adsorbed to them, and thereby forming surface coronas, is limited. However, we show here that the toxic effect of NPs of tungsten carbide cobalt (WC–Co) and cobalt (Co) on the crustacean *Daphnia magna* is postponed in the presence of natural biological degradation products (eco-corona biomolecules). For *Daphnia* exposed to WC–Co NPs the survival time increased with 20–25% and for Co NPs with 30–47% after mixing the particles with a solution of eco-corona biomolecules before exposure. This suggests that an eco-corona, composed of biomolecules always present in natural ecosystems, reduces the toxic potency of both studied NPs. Further, the eco-coronas did not affect the particle uptake, suggesting that the reduction in toxicity was related to the particle-organism interaction after eco-corona formation. In a broader context, this implies that although the increasing use and production of NPs may constitute a novel, global environmental threat, the acute toxicity and long-term effects of some NPs will, at least under certain conditions, be reduced as they enter natural ecosystems.

## Introduction

Nanotechnology has revolutionized our lives during the past decades, but is associated with an increased concern regarding its possible adverse effects on human health and safety^1, 2^. The increased use of nano-sized materials may further pose a risk that nanoparticles (NPs) enter natural ecosystems through accidental spills and insufficient waste handling of products containing nanomaterials, or via diffuse dispersion due to surface wear and material degradation. Despite an accelerating production and use of nano-sized particles, our knowledge of their toxicological properties is still limited when it comes to fundamental nano-specific mechanisms.

A considerable amount of the NPs used in our everyday lives is eventually dispersed via sewage systems and will eventually reach lakes and oceans^3^, although predicted environmental concentrations for engineered NPs such as TiO_2_, Ag, fullerenes, and ZnO are relatively low (µg to pg per L)^4^. Upon environmental contact, biomolecules, such as proteins and various natural biological degradation products, adsorb to the surface of naked nanoparticles forming an eco-corona. This corona alters the surface characteristics and may also amend the biological properties of the NPs, and thereby interactions between the particle and a biological system^5, 6^. Changes in particle morphology, reactivity and stability influence the likelihood that the NPs may bind to biological receptors or are recognized as e.g. food resources by organisms^7^. Currently standardized OECD tests to evaluate the toxic effects of chemicals and nanomaterials are not accounting for the presence of natural organic substances forming coronas on NPs. In order to assess the actual effects of NPs entering natural ecosystems, and thereby provide decision support for society regarding the use of nanomaterials, it is therefore crucial to understand the formation and function of natural organic matter and how they interact with NPs and influence their toxic properties. It has previously been reported that tungsten carbide (WC) NPs are toxic to the freshwater zooplankter *Daphnia magna* at high concentrations (10 mg L^−1^) and long exposure times, leading to shorter lifespan and delayed start of first reproduction among the animals^8^. WC, often cemented with cobalt (Co)^9^, is widely used in modern society, for example in drilling tools and car tire studs, due to its hard metal properties^10^. The use and wear of the material generate smaller particles, of which some reach the nano size range that may be dispersed into the environment^11–13^. Tungsten contamination is distributed widely, partly due to aerial transportation. Airborne concentrations of nano-sized tungsten (< 100 nm) have been estimated to 0.006–1.2 ng/m^3^ in traffic settings^12^, and modelling has suggested that about 1000 kg of nano-sized WC–Co are released in Sweden each year^13^. Studies of ecotoxicological effects from NPs of tungsten carbide cobalt (WC–Co) and Co on aquatic organisms are rare, although WC–Co particles have been shown to be more reactive and generate more reactive oxygen species (ROS) compared with WC^14, 15^. It should, however, be noted that low concentrations of Co are essential for the well-being of most organisms^16, 17^.

Here, we specifically focus on the fate of WC–Co NPs in aquatic ecosystems and hypothesize that the corona, formed at the surfaces of WC–Co through adsorption of natural degradation products, acts like a natural shield that reduces the toxicity of the NPs when they are taken up by organisms. As Co is the binder phase (5 wt%) of the WC particles in the WC–Co NPs, we also performed complementary studies on Co NPs. Natural organic matter, such as humic acid, has in several cases been seen to reduce toxicity of metallic NPs by different mechanisms^18^, such as reduced bioavailability of released metal ions due to complexation. *Daphnia* has previously been shown to secrete proteins into their surroundings, facilitating the formation of a NP corona on polymer NPs^7, 19^. In addition to address environmental effects on *Daphnia* related to the dispersion of WC–Co NPs, a complementary aim of this study is to concentrate and characterize a naturally produced solution of corona forming biomolecules (from now on referred to as eco-corona biomolecules) and to investigate how this eco-corona affects the NPs and their toxic potency. These eco-corona biomolecules were used to condition NPs in long-term exposure experiments with *Daphnia magna*. This approach creates a test system that mimics a natural exposure scenario in a two-level food-chain in a natural ecosystem composed of zooplankton and algae, where their degradation products are natural components of the surrounding environment to which dispersed NPs are exposed when entering the system. Although the chemical composition of an eco-corona may vary with type of water, catchment, and the organism composition in a natural aquatic ecosystem, biomolecules potentially forming eco-coronas around novel objects, including nanoparticles, are always present. Hence, the overall main aim with our study was not to assess the specific chemical composition of the eco-corona, but to assess if it may reduce the toxicity of nanoparticles, which would provide much needed knowledge regarding risk analysis of NP´s as they enter natural ecosystems.

## Results and discussion

**Figure 1:**
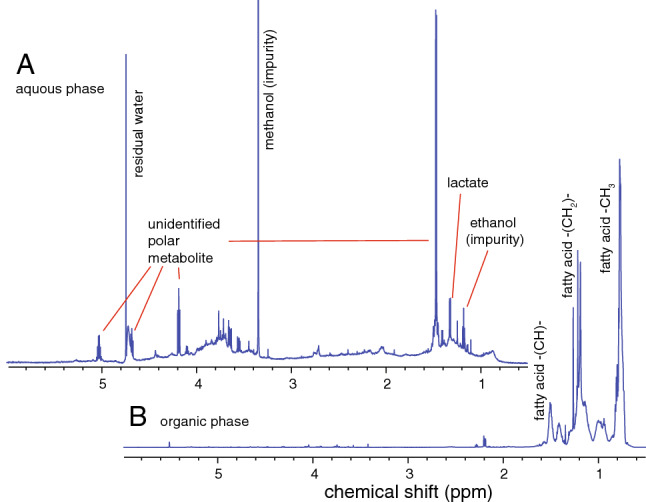
1D ^1^H NMR spectra of the aqueous (**A**) and organic (**B**) fractions after chloroform–methanol-water extraction of the eco-corona solution. (**A**) The aqueous fraction is dominated by lactate and an unknown metabolite. The unknown metabolite consists of a methyl (CH_3_) group connected to a methine (CH) with a hydroxyl (OH) or nitrogen and followed by a methylene (CH_2_) group with an unknown substituent. (**B**) The organic phase is dominated by fatty acids.

When NPs, such as Co and WC–Co, enter natural ecosystems, they encounter naturally occurring organic molecules which can adsorb and form a surface corona on the NPs^20^, potentially altering their toxicity^6^. Such eco-corona biomolecules, in the form of excretion products from *Daphnia* feeding on algae (*Scenedesmus* sp.)*,* were collected and concentrated by ion exchange chromatography. This solution likely contained a mixture of biomolecules from both algae and *Daphnia*. Analysis by means of nuclear magnetic resonance spectroscopy (NMR) could identify some, but not all, metabolites present in the solution. 1D and 2D ^1^H NMR spectra were acquired on the aqueous and organic fractions after chloroform–methanol-water extraction of the eco-corona biomolecule solution. The aqueous fraction was dominated by lactate and an unknown metabolite with at least three carbon molecules: a methyl (CH_3_; δ_C_ = 18.4 ppm, δ_H_ = 1.46 ppm) group connected to a methine (CH; δ_C_ = 75.6 ppm, δ_H_ = 5.02 ppm) with a hydroxyl (OH) or nitrogen and followed by a methylene (CH_2_; δ_C_ = 71.6 ppm, δ_H_ = 4.68, 4.18 ppm) group with an unknown substituent (Fig. 1A). The organic phase was dominated by fatty acids (Fig. 1B).

**Figure 2:**
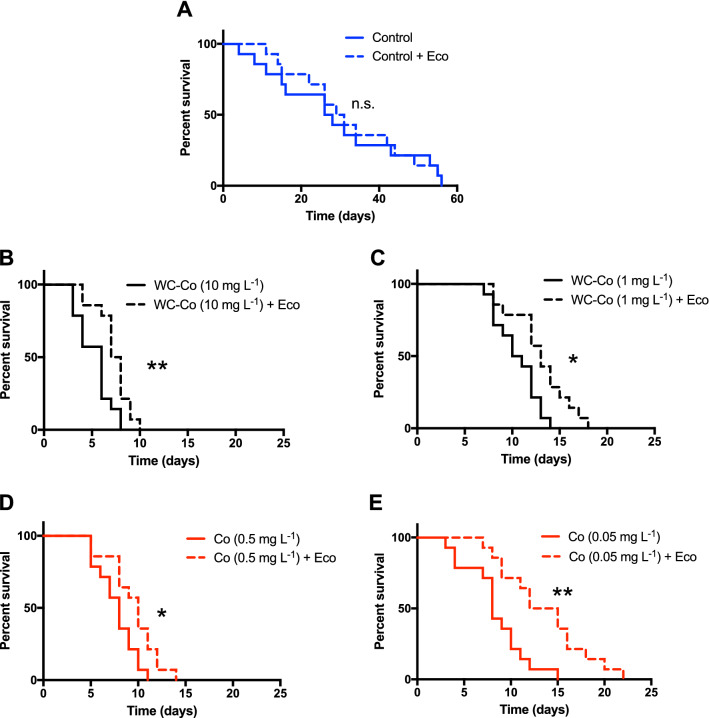
Kaplan–Meier survival curves for *Daphnia magna* with (solid line) and without (dashed line) addition of eco-corona biomolecules. Data is shown for Control (**A**), WC–Co NPs at concentrations of 10 mg L^−1^ (**B**) and 1 mg L^−1^ (**C**), and for Co NPs at concentrations of 0.5 mg L^−1^ (**D**) and 0.05 mg L^−1^ (**E**), corresponding to the 5 wt% Co included in WC–Co. Eco denotes excreted eco-corona biomolecules. One asterisk denotes significant differences at the 5% level and two asterisks at the 1% level, whereas n.s. denotes no significant difference. Each treatment was replicated 14 times.

Next, we investigated if the presence of the environmentally relevant eco-corona biomolecules would interact with the Co and WC–Co NPs forming an eco-corona that would affect their long-term toxicity. *Daphnia* were exposed to pristine Co or WC–Co NPs in solutions with and without the eco-corona biomolecules at a total organic carbon concentration of 0.63 mg L^−1^. The investigated Co NP concentrations were selected to correspond to the relative amount of Co (nominal bulk content, 5 wt%) in relation to WC of the WC–Co NPs. Kaplan–Meier survival analyses revealed that the conditioning of the NPs in a solution containing the eco-corona biomolecules had a significant positive effect, with p-values always above 5%, on *Daphnia* survival for both WC–Co NPs (χ^2^_(1)_ = 8.044, *p* < 0.01; χ^2^_(1)_ = 6.539, *p* < 0.05, high and low concentration respectively) and Co (χ^2^_(1)_ = 5.339, *p* < 0.05; χ^2^_(1)_ = 10.04, *p* < 0.01, high and low concentration respectively) NPs at both the higher and lower exposure concentrations (Fig. 2A–E). Hence, at the high concentration of WC–Co NPs, *Daphnia* survived for 8 days, whereas they survived until day 10 when exposed to NPs preconditioned with the eco-corona biomolecules (Fig. 2B). Similarly, at the lower concentration of the WC–Co NPs, animal survival was 14 and 18 days when exposed to particles without and with eco-corona, respectively (Fig. 2C). Hence, the results clearly show an overall increase in survival of 20–25% upon exposure of WC–Co NPs in the presence of the eco-corona biomolecules (Fig. 2). Similar effects were observed for the Co NPs with 30 and 47% increased survival for the high and low concentrations, respectively (Fig. 2D,E).

**Figure 3:**
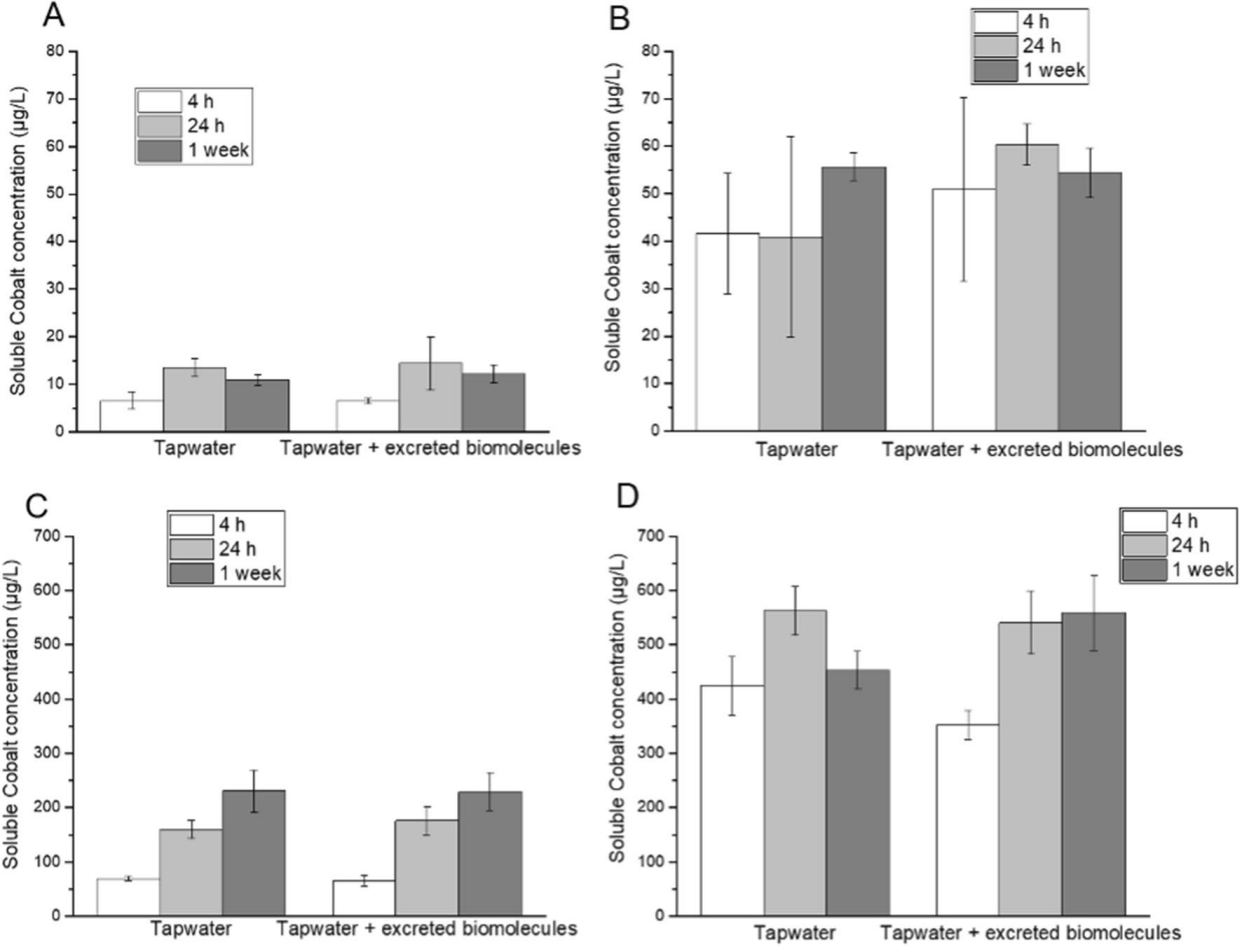
Co release investigated by AAS. (**A**) Co NPs, loading 0.05 mg L^−1^, (**B**) WC–Co NPs, loading 1 mg L^−1^, (**C**) Co NPs, loading 0.5 mg L^−1^ (**D**) WC–Co NPs, loading 10 mg L^−1^. Eco-corona denotes eco-corona biomolecules. The error bars represent a standard deviation as derived from six independent samples.

Our data furthermore show that the survival was concentration dependent with a reduction in survival of several days when increasing the particle concentration from 1 to 10 mg L^−1^ (Fig. 2). In contrast, the addition of the eco-corona biomolecules to the control treatment without NPs had no effect on the survival of the *Daphnia* (χ^2^_(1)_ = 0.0726, *p* > 0.05), and the animals survived for up to 56 days. This shows that even though the presence of the eco-corona biomolecules reduces the toxic effects of WC–Co or Co NPs, the presence of both NPs strongly reduces the survival of *Daphnia* (Fig. 2). Several potential mechanisms may explain the observed protection induced by the eco-corona biomolecules^18^ of which three will be discussed below; (i) the eco-corona biomolecules may influence the Co dissolution from the Co- and WC–Co NPs^21^ and modulate the released Co ion concentration in solution and hence the toxic response, (ii) biomolecules forming the eco-corona may change the bioavailability of dissolved Co in solution, and (iii) adsorption of eco-corona biomolecules on the NPs may change the surface characteristics and how the particles interact with the zooplankton. The first hypothesis, stating that the dissolution of Co is influenced by the presence of the eco-corona biomolecules, was addressed by assessing differences in the release of Co from Co and WC–Co NPs with and without conditioning in the eco-corona biomolecule solution. No measurements were performed on released W-ions due to their relatively slow release^21^ and low toxicity (EC50 value of approx. 89 mg L^−1^ for W^6+^^22^) compared with Co (water quality guideline of 8 µg L^−1^ Co for chronic exposure in freshwater)^16^. The concentration of released Co from the Co NPs increased during the first 24 h but did not significantly increase further for any of the two particle concentrations investigated (Fig. 3). For the WC–Co NPs, the release of Co was rapid with concentrations close to the nominal amount of Co (50 µg L^−1^) already after 4 h for the lower particle concentration (1 mg L^−1^). For the higher particle loading (10 mg L^−1^), an increase in Co release was observed after 4 h in tap water and after 24 h and 1 week in the presence of the eco-corona biomolecules. Observed release rates are in line with previous studies on release rates of Co from WC–Co in surface water, displaying a fast release of most Co during the first 4 h of exposure^21^.

**Figure 4:**
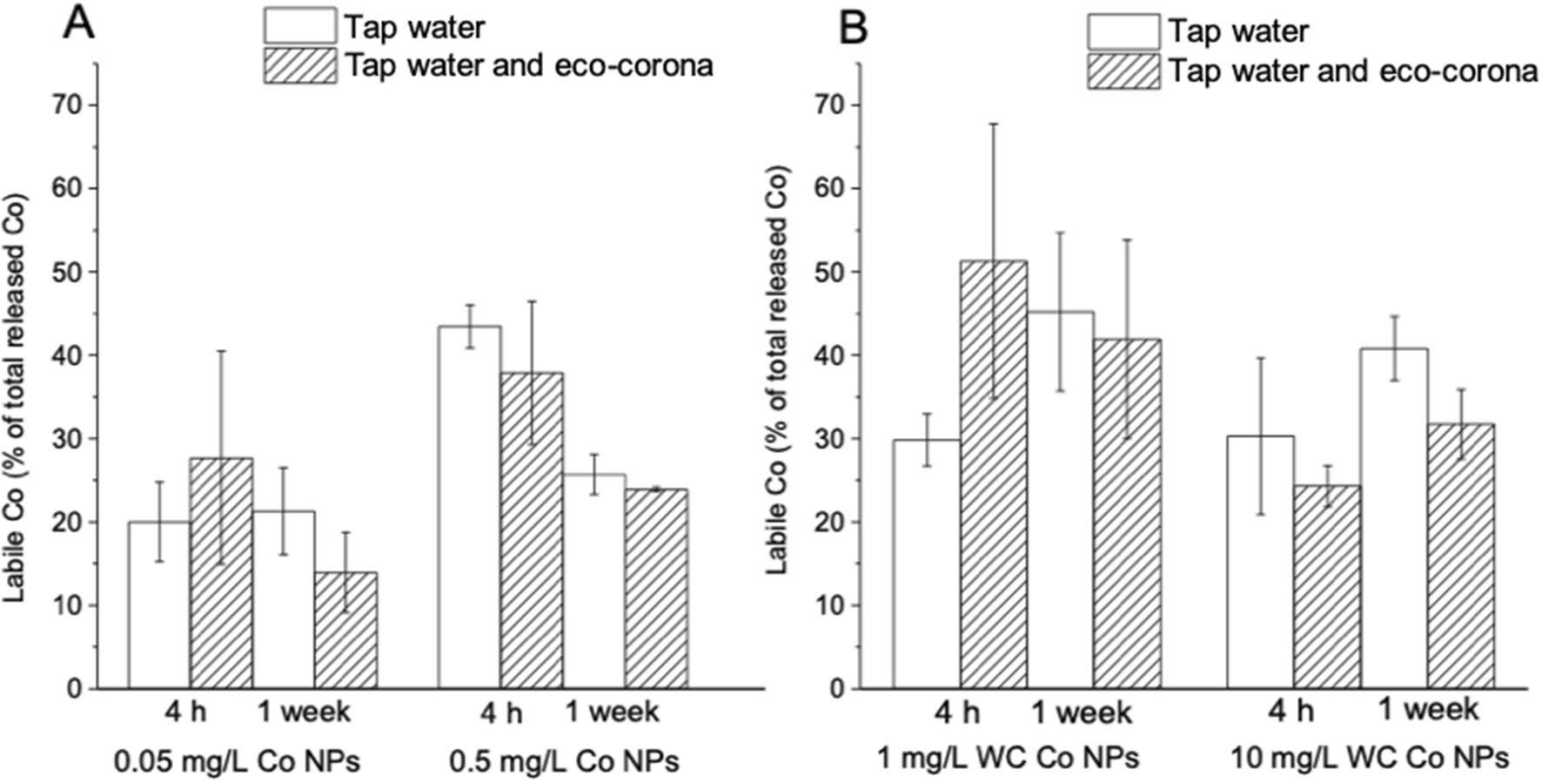
Percentage of labile Co as deduced by the DGT method, following NP separation from solution. The percentage is the fraction of the released Co in Fig. 3 which is detected by DGT and assumed to be bioavailable. (**A**) Co NPs, loading 0.05 mg L^−1^, (**B**) Co NPs, loading 0.5 mg L^−1^, (**C**) WC–Co NPs, loading 1 mg L^−1^, (**D**) WC–Co NPs, loading 10 mg L^−1^. Eco-corona denotes eco-corona biomolecules. The error bars represent a standard deviation as derived from three independent samples.

The results from a two-way ANOVA analysis using the time and type of solution as fixed factors are presented in Supplementary Table S2. The presence of the eco-corona biomolecules did not lead to any significant differences in released amounts of Co compared with tap water only, as seen in p-values > 0.05 for the solution factor for all NPs and concentrations. The hypothesis that the presence of the eco-corona biomolecules results in differences in Co dissolution was therefore rejected. The second hypothesis, stating that the eco-corona biomolecules change the bioavailability of dissolved Co, was addressed by investigating the labile fraction of released Co from the Co and WC–Co NPs using the Diffusive Gradients in Thin-film (DGT) method. The labile Co fraction reflects Co species that can easily dissociate and thus react with biological ligands of aquatic organisms, predominantly free ions and inorganic complexes, as well as complexes with small organic molecules, and is therefore an estimate of the potentially bioavailable fraction of the total amount of released Co^23^. The non-labile fraction mainly consists of released Co that is strongly associated to particulate matter or organic molecules, such as humic acid less prone to dissociate and hence not bioavailable^23^. Figure 4 shows the labile fraction of Co in tap water with and without the eco-corona biomolecules. Supplementary Table S3 presents results from two-way ANOVA analyses using the time and type of solution as fixed factors. The lack of a significant difference between the labile fraction of Co in tap water with and without eco-corona biomolecules seen in Supplementary Table S3 (p > 0.05 for the solution factor for all NP) implies that the reduced toxicity of Co and WC–Co NPs in the presence of eco-corona biomolecules (Fig. 2) was not directly related to differences in bioavailability (labile fraction) of released Co species. Hence, there is no support for the hypothesis that the presence of the eco-corona biomolecules changes the bioavailable fraction of released Co. However, we cannot rule out this hypothesis completely, since higher particle loadings shows a tendency to reduce the bioavailable fraction with time in the presence of the eco-corona biomolecules for both the Co and the WC–Co NPs (although not statistically significant). The results nevertheless show that the total concentration of released Co is not an accurate predictor of the bioavailable fraction as the labile fraction was 10–50% of the total amount of the released Co in the case of the Co NPs and between 20 and 65% for the WC–Co NPs.

**Figure 5:**
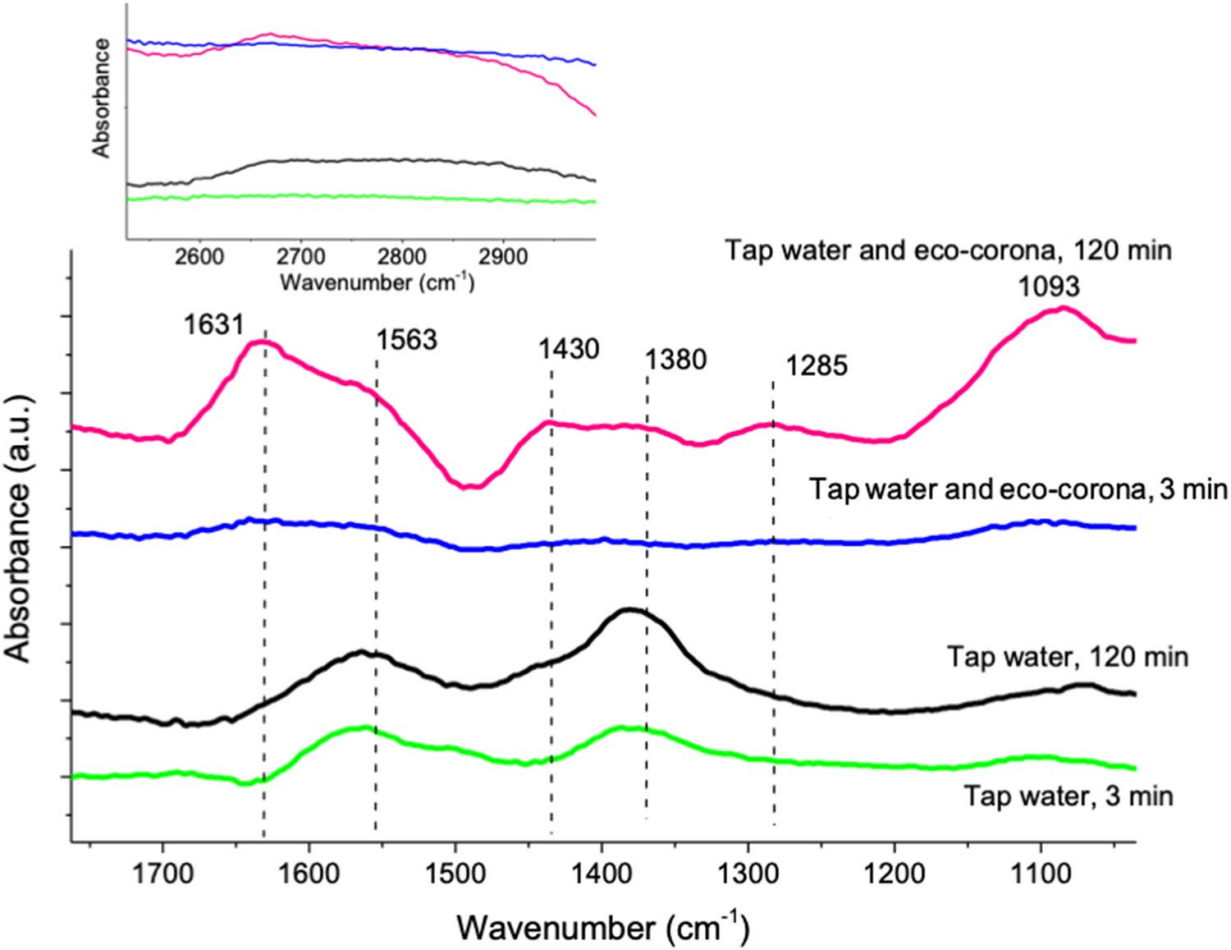
ATR-FTIR spectra for Co NPs in tap water, with and without eco-corona biomolecules, after 3 and 120 min. The inset shows the CH-stretching region for the same spectra. The spectra have been offset for clarity.

**Figure 6:**
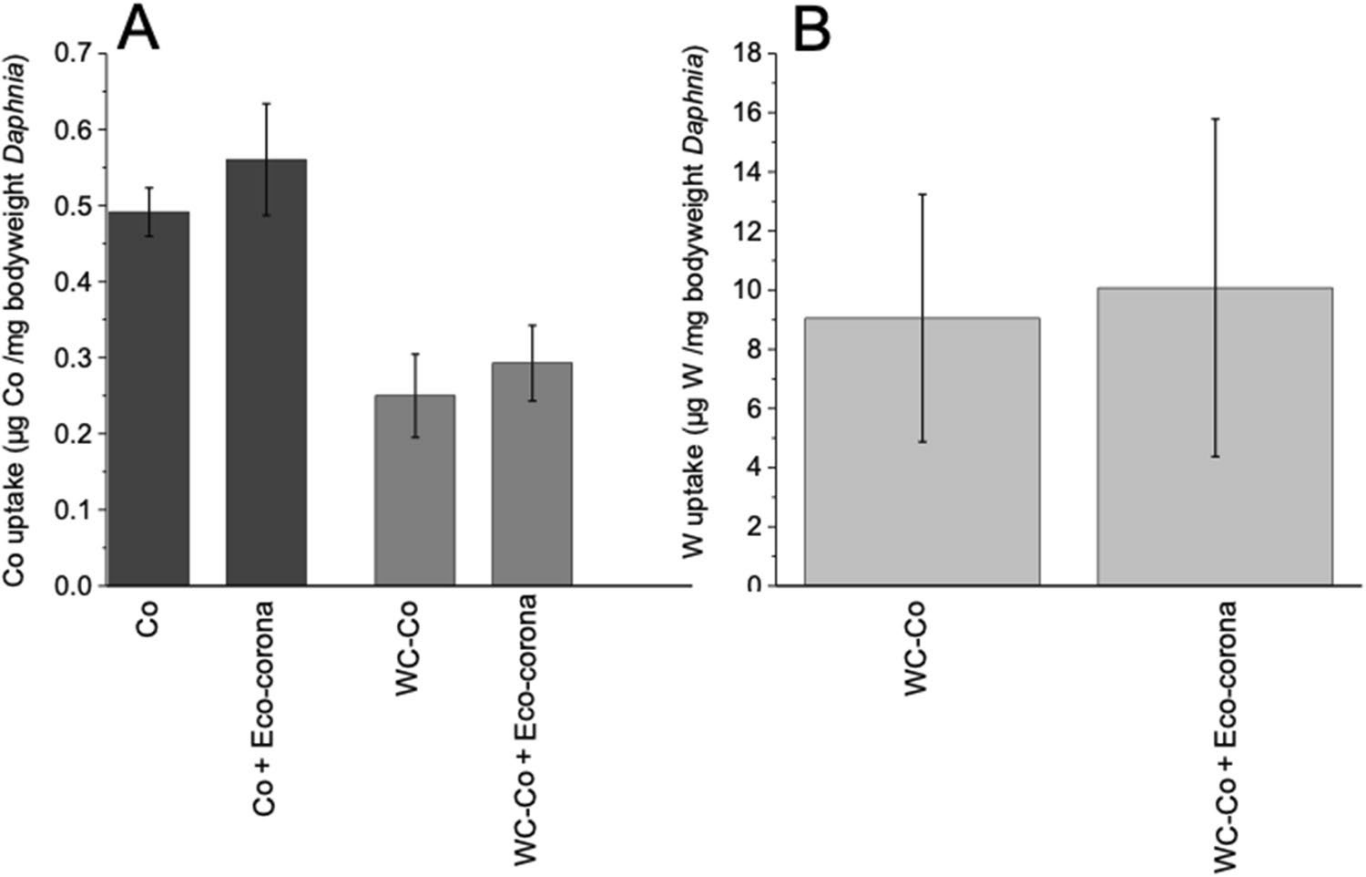
Uptake of Co (**A**) and W (**B**) by *Daphnia* exposed to Co and WC NPs with and without eco-corona biomolecules during 48 h. The error bars represent a standard deviation as derived from five independent samples.

The third hypothesis addressed if the adsorption of eco-corona biomolecules on the surface of the NPs influences their interaction with zooplankton. These aspects were studied by investigating the potential adsorption of the eco-corona biomolecules forming a corona on the Co and WC–Co NPs surfaces by means of ATR-FTIR and its possible effects on the zeta-potential (apparent surface charge). ATR-FTIR results are shown in Fig. 5 for the Co NPs only as no adsorption was observed for the WC–Co NPs in tap water with and without the eco-corona biomolecules. The bands observed at 1380 and 1563 cm^−1^ on the Co NPs in tap water were tentatively attributed to Co-carbonates^24^, a result of the relatively high carbonate content of the tap water (annual local average HCO_3_ concentration: 50 mg L^−1^, June 2017–June 2018^25^). Additional bands at 1430 and 1631 cm^−1^ are related to the adsorption of the eco-corona biomolecules to the Co NPs. These bands are likely assigned to carboxylates associated with fatty acids^26^ and lactate, identified components by means of NMR of the eco-corona (see Fig. 1B), or humic-like substances^27, 28^. The absence of any detectable bands in the CH-stretching region implies short-chained fatty acids. If adsorbed as long chained fatty acids, strong signals from CH-groups would be present in that spectral region, as in the case of for example oleic acid with 18 carbons^26^. Additional bands at 1093 cm^−1^ and 1285 cm^−1^ on the Co NPs in the presence of the eco-corona biomolecules are likely associated with humic-like substances or biomolecules from phenol C–O stretches (1093 cm^−1^^27, 28^) or C–O stretches of polysaccharides from algae^29^. These humic-like substances may originate from the degradation of algae during the preparation of the eco-corona biomolecule solution. Even though adsorption of different biomolecules was evident for the Co NPs (not observed for the WC–Co NPs), their presence had no significant impact on the extent of released Co (Fig. 3) from the NPs. Any initial adsorption of eco-corona biomolecules of the WC–Co NPs cannot be excluded due to the low amount of Co (5 wt%) between the WC particles that rapidly was dissolved into solution (Fig. 3). Differences in zeta potential of the Co and WC–Co NPs were estimated in tap water with and without the eco-corona biomolecules (Supplementary Fig. S2). The Co NPs revealed a bimodal distribution in tap water with peaks at − 19 mV and − 3 mV that changed to a single peak at − 9 mV in the presence of the eco-corona biomolecules. No difference in zeta potential (− 15 mV) was observed for the WC–Co NPs by the presence of the eco-corona biomolecules. The results corroborate the ATR-FTIR results as the slight change in zeta potential correlates with the adsorption of the eco-corona biomolecules on the Co NPs whereas no, or minor, adsorption or change in zeta potential was evident in the case of the WC–Co NPs. Hence, this hypothesis was corroborated for the Co NPs as the adsorption of eco-corona biomolecules formed a corona on the surface of the Co NPs and that these interactions most likely influenced their interaction with zooplankton and prolonged the survival time of *Daphnia*. Studies on NP size in solution using dynamic light scattering (PCCS) showed extensive agglomeration, which unfortunately prohibited quantification of the hydrodynamic size in solution of the studied NPs. The agglomeration was extensive, implying presence of micro-sized agglomerates, also with the addition of excreted biomolecules. The eco-corona on the NPs could thus not colloidally stabilize the NPs. Changed colloidal stability would influence the exposure of the NPs to the *Daphnia* and potentially subsequent toxicity, for example seen from increased toxicity with increased agitation for WC NPs^8^. The uptake study showed no differences in the amount of W and Co taken up by the organisms in the different treatments (Student’s t-test, p > 0.05, Fig. 6). This also corroborates the conclusion that the reduced toxicity is due to that the eco-corona influences how the particle interacts with the organism. Previous studies on polystyrene and PVP capped metal oxide NPs have shown increased toxicity of NPs once conditioned with proteins secreted from *Daphnia,* effects attributed to higher particle uptake^7, 30^. Interestingly, we here observed reduced toxicity of the NPs in a long-term study when conditioned with eco-corona biomolecules. However, the formation of NP coronas has been shown to inflict both positive and negative effects on toxicity and it is likely that they are both corona and material specific^6, 18, 31^. No tendency for acute (24–48 h) toxic effects on *Daphnia* was observed in our study, suggesting that the effect of the initial conditioning of the WC–Co and Co NPs with the eco-corona biomolecules had lasting effects despite the continuous build-up of secreted degradation products throughout the experiment in all treatments.

We conclude that neither any significant changes in dissolution of Co, nor in Co speciation, were influenced by the presence of the eco-corona biomolecules that could explain the observed reduction in NP toxicity towards *Daphnia*. The hypothesis that the eco-corona biomolecules would adsorb on the NP surface forming a corona and influence the interactions between zooplankton and NPs was, however, corroborated even though no evident adsorption or effect on the zeta potential were observed for the WC–Co NPs in the presence of the eco-corona biomolecules. However, as the nominal Co content of the WC–Co NPs was only 5 wt% and that adsorption predominantly took place on Co (the binder between the WC particles), and not on the WC particles^32^, it is very likely that the extent of adsorption was too small to be detected using the ATR-FTIR technique. Reduced toxicity on *Daphnia* induced both by the Co NPs and the WC–Co NPs might be explained by a reduced generation of ROS in the presence of natural organic matter^18^. However, these detailed mechanistic aspects deserve further attention in future studies.

In a broader context our results imply that environmental hazards from these types of NPs are reduced as the particles enter natural ecosystems due to the presence and surface interactions of biological degradation products such as the eco-corona biomolecules. Hence, compared to standard laboratory toxicity tests typically performed in the absence of any eco-corona biomolecules, our study provides a more accurate and environmentally relevant understanding on how the toxicity of nanomaterials may affect natural ecosystems.

## Materials and methods

### Nanoparticles and dispersion

Cemented tungsten carbide cobalt (WC–Co) NPs were obtained from American Elements (product code WC-CO-03MNP.200N; LOT # 1801223479-400, < 200 nm, 40–80 nm) containing 5 wt% Co and a purity of 99.9% (metal basis). The Co NPs were also purchased from American Elements (product code CO-M-028M-NP.100N, LOT # 1211392979-814, < 100 nm) with a purity of 99.8% (metal basis). These NPs have been thoroughly characterized elsewhere, and their main characteristics are summarized in Supplementary Table S1, see further^21, 32, 33^. Their particle size and morphology, based on transmission electron microscope (TEM) measurements, are presented in Supplementary Fig. S1. The NPs were dispersed by ultrasonication using a probe sonicator (Branson Sonifier 250, Ø 13 mm, 400 W output power, 20 kHz). The calibration of the acoustic energy delivered during sonication was based on established protocols for NP dispersion^34^. It involves a calorimetric method to calibrate the delivered acoustic energy by adjusting the probe sonicator amplitude. For the probe used in this study, a 40% sonication amplitude (50% duty cycle) during 10 min was used. These settings correspond to a delivered acoustic energy of 8200 J (for details see^34^). 6 mL MilliQ water was added to the vials (WHEA986581, Wheaton Industries Inc., USA). The glass vials were positioned in an ice-filled bowl with the sonication probe inserted between the upper quarter and upper half of the solution in the vial. NP dispersions from these stock solutions were then diluted to reach the desired concentrations (10 mg L^−1^ and 1 mg L^−1^ for the WC–Co NPs and 0.5 mg L^−1^ and 0.05 mg L^−1^ for the Co NPs) and used directly after the dispersion. Toxic effects on *D. magna* have been shown for WC NPs at 10 mg L^−1^*.* We therefore chose to use this concentration and also assess the effects with a 10 times dilution of the WC concentration. Co concentrations were based on the 5 wt% of Co in WC–Co. Tap-water (300 times dilution, resulting in 0.63 mg L^−1^ TOC) was used to disperse the eco-corona biomolecule solution.

### Preparation of the eco-corona biomolecule solution

The eco-corona biomolecules were prepared by adding 10 L each of a mixture of tap water and an algal culture dominated by the green algae *Scenedesmus* sp. into two aquaria. The final algal concentration was set to 450 µg chl-a L^−1^ using an AlgaeLabAnalyser (bbe Moldaenke GmbH, Germany). After adding the algal solution, we added 500 juvenile *Daphnia magna* into each aquarium and left them for two weeks at 18 °C with a photoperiod of 12 h/12 h light/dark conditions to allow the *Daphnia* to ingest most of the algae. After the ingestion period the solution was filtered through a Whatman GF/C filter (GE Healthcare Life Sciences) to remove *Daphnia*, remaining algae and debris. After filtration, the solution from the two aquaria was pooled into a 20 L vessel and 25 mL of a strong anionic exchanger, diethylaminoethyl sepharose (DEAE-sepharose), was added. The anionic exchanger was left in the solution for 24 h during continuous mixing. After 24 h, the anionic exchanger was filtered out, collected in an elution column, and the bound material was eluted with 1 M NaCl. After elution, the eco-corona biomolecule solution was loaded in a dialyze tube with a molecular weight cut-off of 6000–8000 Da (Spectra/Por Dialysis Membrane, order nr: 132660) and dialyzed against distilled water during 72 h. The water was exchanged twice during the first 24 h of dialysis and then once per day for the remaining 2 days. After dialysis, the eco-corona biomolecule solution was transferred into 1 mL aliquots and stored at − 20 °C. The concentration of total organic carbon (TOC) in the eco-corona biomolecule solution was analyzed using a Shimadzu TOC-V CPN Total Organic Carbon Analyzer.

### NMR characterization of the eco-corona biomolecules

5 mL of eco-corona biomolecule solution was freeze dried and extracted in chloroform–methanol-water according to Beckonert et al.^35^. The aqueous fraction was freeze dried and the organic fraction was dried after which both fractions were stored at − 80 °C. Prior to the NMR measurements, the aqueous sample was rehydrated in 200 µL of 50 mM phosphate buffer (pH 7.4) in D_2_O, and the organic fraction in 200 µL CDCl_3_. The solutions contained the chemical shift reference DSS (4,4-dimethyl-4-silapentane-1-sulfonic acid) and sodium azide, and TMS (tetramethylsilane), respectively. Measurements were performed at 25 °C using a Bruker Avance-III 700 spectrometer (Bruker Biospin, Germany) equipped with a double tuned ^1^H–^13^C cryoprobe and run at a ^1^H frequency of 700.20 MHz. The ^1^H NMR spectra were acquired using a single 90° pulse experiment. Two-dimensional ^1^H–^1^H COSY, ^1^H–^1^H TOCSY, ^1^H–^13^C HSQC, ^1^H–^13^C HSQC-TOCSY and ^1^H–^13^C HMBC spectra were acquired for assignment purposes.

### Eco-corona formation

The adsorption of eco-corona biomolecules to the Co and WC–Co NPs leading to the formation of an eco-corona was investigated as described in Mei et al*.*^36^ using a Bruker Tensor 37 FTIR Spectrometer. A Platinum ATR-FTIR accessory was used equipped with a diamond crystal and an angle of incidence for the IR beam of 45°. 256 scans, collected at a resolution of 4 cm^−1^, were co-added for each spectrum. The Co NPs were dispersed as described above, with the difference that the medium was ethanol and that the particle loading was 2.5 g L^−1^ in the sonicated stock solution. A total volume of ca. 50 µL was drop-cast onto the ATR crystal. The film was then left for 2 h before the measurements. After that, an ATR-IR flow accessory was put onto the film and MilliQ water was used to rinse the system to remove any loosely attached NPs. A spectrum of the particle film and MilliQ water was used as background for the studied NP solutions of interest.

### Zeta potential

The zeta potential was investigated using a Malvern Zetasizer Nano Z instrument.

Three independent samples were tested and each sample was measured twice, directly after each other. The samples were prepared using the same sonication method as described above. The Smoluchowski approximation was used for calculating the zeta potential from the electrophoretic mobility.

### Particle size in solution

Photon cross correlation spectroscopy (PCCS) was used to investigate the hydrodynamic particle size. The measurements were conducted on a Nanophox (Sympatec GmbH, Germany), at room temperature, using Eppendorf cuvettes (Eppendorf AG, Germany, UVette Routine pack).

### Metal release

Metal release measurements was adapted from Mei et al*.*^36^. Three independent samples were repeated twice, generating six measurements for each exposure condition. A blank sample without any NPs was prepared in parallel. The replicates and the blank sample were incubated at 20 ± 1 °C (no agitation) for up to 1 week. A new stock solution was prepared just before the exposure. Following incubation, 5 mL of each replicate was pipetted to an acid-cleaned plastic tube and another 5 mL was filtrated through an inorganic membrane filter (0.02 µm, 25 mm diameter, Anotop 25 syringe filter, GE Healthcare Life Sciences) to an acid-cleaned plastic tube. 5 mL of the parallel exposed blank sample was filtrated in the same way. The samples were acidified to pH < 2 with puriss p.a. 65% HNO_3_ and stored at room temperature prior to analysis. The filtration procedure was checked to ensure no Co ion retention or contamination (AAS), as well as complete loss of NPs from solution (Nanoparticle Tracking Analysis).

Graphite furnace- or flame atomic absorption spectroscopy (AAS, Perkin Elmer AAnalyst 800) was used to measure total Co concentrations in the solution. Calibration standards were prepared from a 1 g L^−1^ (Perkin Elmer) in 1% HNO_3_. Each sample was measured thrice, and the resulting average concentration was used, after subtraction of blank samples. The detection limit was estimated as three times the standard deviation of the blank and the determination limit was ten times the standard deviation of the same blank. This resulted in detection and determination limits of 1.5 and 5 µg L^−1^, respectively.

### Co bioavailability

The bioavailable (labile) fraction of Co was estimated using the diffusive gradient in thin-films (DGT) technique, as described in Zhang and Davison^37^. DGT is an in situ technique that is comprised by three layers held together by a plastic casing. The first layer is a membrane filter with a 0.45 µm pore size. The second layer is made up of an ion-permeable gel membrane that only permits diffusion of labile metal species, and the third layer is an ion-exchange resin designed to selectively accumulate metal ions. After exposure of the DGT plastic holder to the solution of interest, the resin is removed and the metal is eluted in 1 M HNO_3_ during 24 h. In this study, the DGT holders were placed in solutions from the metal release studies, e.g. after 20 nm filtration (see above), for a time period of 24 h. The bioavailable concentration was calculated using the established formula using Fick’s law and a constant for Co diffusion, and the volume of the Chelex resin absorbing the Co. The DGT-available concentration in the tap water, with and without the eco-corona biomolecules, was calculated using Eq. (1).

1 $$C_{DGT} = \frac{{C_{sol} \left( {V_{{HNO_{3} }} + V_{gel} } \right) \times b}}{{f_{e} DtA}}.$$

Here, *C*_*sol*_ is the determined concentration of the eluate (g L^−1^), *V*_*HNO3*_ is the volume of the eluate (L), *V*_*gel*_ is the volume of the resin gel (L), b is the thickness of the diffusive gel and the membrane (cm), *f*_*e*_ is the elution factor (-), *D* is the diffusion coefficient (cm^2^ s^−1^), *t* is the time (s) and *A* is the area of the membrane (cm^2^).

Only the free ion and labile species permeate the membrane and accumulate in the resin. Thus, the Co concentration measured by DGT correlates well with the bioavailable Co concentration.

### Toxicological assay

To evaluate how the presence of the collected eco-corona biomolecules affected the NP toxicity we exposed *Daphnia magna* to two concentrations (10 and 1 mg L^−1^) of WC–Co NPs with and without addition of eco-corona biomolecules. The total Co content of WC–Co NPs corresponds to 5 wt% and has previously been shown to be released within hours once the particles are suspended in solution^21^. Experiments were therefore also performed with Co NPs in concentrations corresponding to the bulk content of the WC–Co particles (i.e. 0.5 and 0.05 mg L^−1^) both with and without addition of eco-corona biomolecules. In order to approach an as natural situation as possible, we reared *Daphnia* (2–3 days old) originating from a natural *Daphnia* population sampled in Lake Bysjön, southern Sweden. Animals were placed into individual 100 mL glass jars containing NPs in a media composed of 70 mL tap water and 10 mL of an algal suspension dominated by *Scenedesmus* sp. The eco-corona solution was added to the media resulting in a concentration corresponding to a 300 times dilution compared to the original solution, corresponding to 0.63 mg L^−1^ TOC. A control was prepared as described above but without the addition of NPs. The experiment was monitored daily to assess the survival of the *Daphnia* and re-suspend the media by gentle mixing using a disposable Pasteur pipette. Any offspring produced was removed each day during the daily monitoring. Due to the short lifespan in relation to the generation time of *D. magna* (8–10 days) in the particle treatments we did not make any further comparisons with respect to reproduction. Food was added twice per week by adding an algal suspension, also compensating for the volume lost through evaporation. Each treatment was replicated 14 times and the experiment ended when all *Daphnia* had died.

### Uptake of WC–Co and Co in *Daphnia magna*

The uptake of WC–Co and Co by *Daphnia magna* was evaluated in the presence and absence of the eco-corona biomolecules for the highest exposure concentrations i.e. 10 and 0.5 mg L^−1^ for WC–Co and Co respectively. The 80 mL of exposure medium was prepared in 100 mL glass jars as described above for the toxicological assay. Ten *Daphnia* were then added to each jar and the organisms were exposed for 48 h. After exposure, all animals were trapped on a 100 µm mesh filter and washed with ultrapure water to remove any particles remaining in the water adhered to the animals. Following the washing procedure, all animals were freeze dried and stored at − 20 °C while awaiting metal analysis. A total of five replicates, each containing ten *Daphnia*, were prepared and analyzed for each treatment. The amount of WC taken up was assessed by analyzing the metal content of tungsten (W) in each sample using inductively coupled mass spectroscopy (ICP-MS). The Co concentration in the *Daphnia* tissue was analyzed using atomic absorption spectroscopy (AAS). The *Daphnia* were digested prior to AAS analysis in 1.5 mL of H_2_O_2_, 1.5 mL of 0.2% NaOH, and 3 mL of MilliQ water in a Metrohm 705 UV Digester for 15 min^21^. All concentrations were normalized according to the dry weight of the organisms, which was calculated using a theoretical length–weight relationship according to Bottrell et al*.*^38^.

### Data analysis

The NMR spectra were processed using iNMR (www.inmr.net) and the spectra were referenced to the DSS or TMS signals at 0 ppm. *Daphnia* survival data was evaluated using Kaplan–Meier survival analysis in GraphPad Prism, version 7e in order to evaluate if the addition of eco-corona biomolecules had any effect on survival. Between treatment differences in the amount of Co released, the percentage of bioavailable Co was evaluated using two-way ANOVAs and the amount of W and Co taken up by *Daphnia* was evaluated using Student’s t-test.

## Supplementary Information

Supplementary Information.

## Data Availability

Raw data are made available upon request.
