# Supplementary material for: Adsorption of bio-organic eco-corona molecules reduces the toxic response to metallic nanoparticles in Daphnia magna
Source: Sci Rep. 2021 May 24;11:10784. doi: 10.1038/s41598-021-90053-5 (PMC8144400; doi:10.1038/s41598-021-90053-5)
Supplement: Supplementary file 1 — Supplementary Information. [file 41598_2021_90053_MOESM1_ESM.pdf]

Supplementary information:

Adsorption of bio-organic eco-corona molecules reduces the toxic response to metallic nanoparticles in *Daphnia magna*

Mikael T. Ekvall, Jonas Hedberg, Inger Odnevall Wallinder, Anders Malmendal, Lars-Anders Hansson, Tommy Cedervall

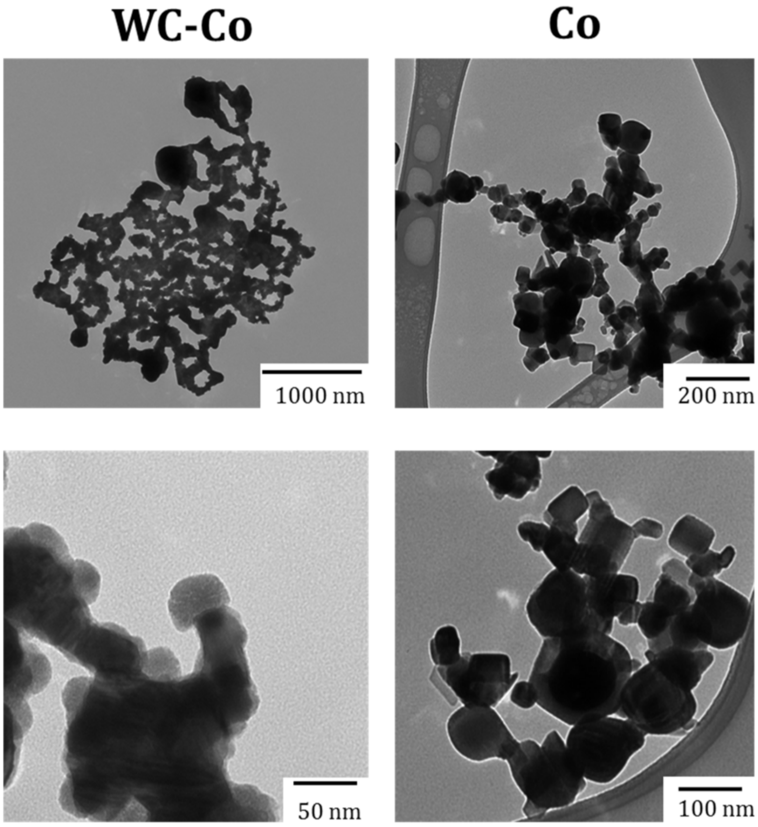

Supplementary Figure S1: TEM (transmission electron microscopy) images of pristine WC-Co and Co NPs (pictures from 21)

Supplementary Table S1: Characteristics of the Co and WC-Co NPs, including particle size ranges (based on TEM), BET (Brunauer Emmet Teller) surface area, and surface oxide composition.

	Co NPs	WC-Co NPs
**Primary size (nm)**	15-35	30-100
**BET surface area (m^2/g)**	10.7±0.4	2.67±0.03
**Surface oxide**	CoO, Co_3O_4	W-Co-O, WO_3

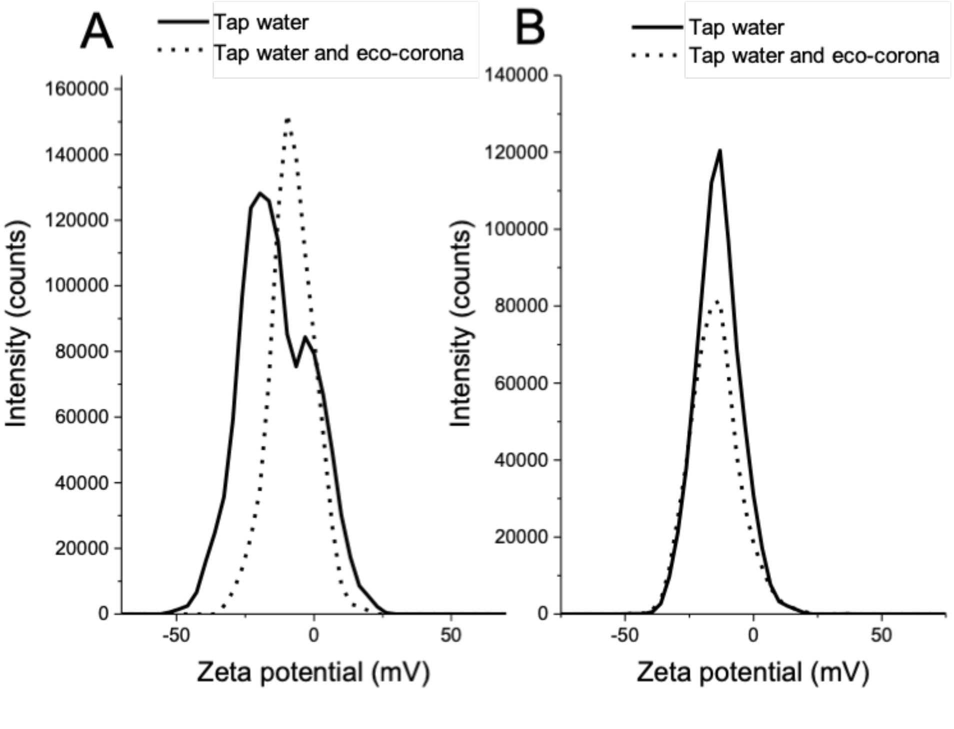

Supplementary Figure S2: Zeta potential distributions of Co NPs (A) and WC-Co NPs in tap water, with and without eco-corona biomolecules (0.63 mg L^{-1} TOC) (B). All spectra show average findings for three independent samples.

Supplementary Table S2. Two-way ANOVA results for the release of Co from Co and WC Co NPs. *df* is the degrees of freedom, *F* is the F factor.

	WC Co NPs, 1 mg/L			WC Co NPs, 10 mg/L			Co NPs, 0.05 mg/L			Co NPs, 0.5 mg/L		
	**df**	**F**	**p-value**	**df**	**F**	**p-value**	**df**	**F**	**p-value**	**df**	**F**	**p-value**
**Time**	2	0.71	0.51	2	17.79	2.57E-04	2	15.47	4.8E-04	2	73.19	1.89E-07
**Solution**	1	1.48	0.25	1	0.02	0.88	1	1.63	0.23	1	0.13	0.73
**Interaction**	2	0.57	0.58	2	5.20	0.02	2	0.50	0.62	2	0.34	0.72

Supplementary Table S3. Two-way ANOVA results for the release of labile Co released from Co and WC and Co NPs. *df* is the degrees of freedom, *F* is the F factor.

	WC Co NPs, 1 mg/L			WC Co NPs, 10 mg/L			Co NPs, 0.05 mg/L			Co NPs, 0.5 mg/L		
	**df**	**F**	**p-value**	**df**	**F**	**p-value**	**df**	**F**	**p-value**	**df**	**F**	**p-value**
**Time**	1	4.50	0.07	1	1.47	0.26	1	0.45	0.52	1	30.18	5.78E-04
**Solution**	1	1.65E-03	0.97	1	2.60	0.15	1	0.02	0.90	1	0.36	0.57
**Interaction**	1	0.65	0.44	1	0.08	0.79	1	0.12	0.74	1	1.15	0.31
